# Predicting Prognosis of Breast Cancer Patients with Brain Metastases in the BMBC Registry—Comparison of Three Different GPA Prognostic Scores

**DOI:** 10.3390/cancers13040844

**Published:** 2021-02-17

**Authors:** Kerstin Riecke, Volkmar Müller, Rudolf Weide, Marcus Schmidt, Tjoung-Won Park-Simon, Volker Möbus, Christoph Mundhenke, Arkadius Polasik, Kristina Lübbe, Tobias Hesse, Elena Laakmann, Marc Thill, Peter A. Fasching, Carsten Denkert, Tanja Fehm, Valentina Nekljudova, Julia Rey, Sibylle Loibl, Isabell Witzel

**Affiliations:** 1 Department of Gynecology, University Medical Center Hamburg-Eppendorf, Martinistraße 52, 20246 Hamburg, Germany; k.riecke@uke.de (K.R.); v.mueller@uke.de (V.M.); e.laakmann@uke.de (E.L.); 2 Oncological Outpatient Department, Neversstraße 5, 56068 Koblenz, Germany; weide@onkologie-koblenz.de; 3 Department of Gynecology, University Medical Center of the Johannes Gutenberg University Mainz, Langenbeckstr. 1, 55131 Mainz, Germany; Marcus.Schmidt@unimedizin-mainz.de; 4 Department of Gynecology, Hannover Medical School, Carl-Neuberg-Straße 1, 30625 Hannover, Germany; Park-Simon.Tjoung-Won@mh-hannover.de; 5 Department of Hematology and Oncology, University Hospital Frankfurt am Main, Theodor-Stern-Kai 7, 60590 Frankfurt am Main, Germany; Volker.Moebus@kgu.de; 6 Clinic Bayreuth, Preuschwitzer Str. 101, 95445 Bayreuth, Germany; christoph.mundhenke@klinikum-bayreuth.de; 7 Department of Gynecology and Obstetrics, University Medical Center Schleswig-Holstein, Arnold-Heller-Straße 3, 24105 Kiel, Germany; 8 Department of Gynecology and Obstetrics, University Medical Center Ulm, Prittwitzstr. 43, 89075 Ulm, Germany; arkadius.polasik@uniklinik-ulm.de; 9 Breast Center, Diakovere Henriettenstift, Schwemannstraße 17, 30559 Hannover, Germany; Kristina.Luebbe@diakovere.de; 10 Department of Gynecology, Agaplesion Diakonie Clinic Rotenburg, Elise-Averdieck-Straße 17, 27356 Rotenburg, Germany; Hesse@diako-online.de; 11 Department of Gynecology and gynecologic Oncology, Agaplesion Markus Hospital, Wilhelm-Epstein-Str. 4, 60341 Frankfurt am Main Frankfurt, Germany; Marc.Thill@fdk.info; 12 Department of Gynecology and Obstetrics, University Hospital Erlangen, Comprehensive Cancer Center Erlangen-EMN, Friedrich-Alexander University Erlangen-Nuremberg, Universitätsstraße 21-23, 91054 Erlangen, Germany; Peter.Fasching@uk-erlangen.de; 13 Institute of Pathology, University Hospital Marburg, Baldingerstraße, 35043 Marburg, Germany; carsten.denkert@uni-marburg.de; 14 Department of Gynecology and Obstetrics, University Hospital Düsseldorf, Moorenstr. 5, 40225 Düsseldorf, Germany; Tanja.Fehm@med.uni-duesseldorf.de; 15 German Breast Group, Martin-Behaim-Straße 12, 63263 Neu-Isenburg, Germany; Valentina.Nekljudova@gbg.de (V.N.); Julia.Rey@gbg.de (J.R.); Sibylle.Loibl@gbg.de (S.L.)

**Keywords:** brain metastases, breast cancer, prognostic scores

## Abstract

**Simple Summary:**

The incidence of brain metastases from breast cancer is increasing and the treatment is still a major challenge. Several scores have been developed in order to estimate the prognosis of patients with brain metastases by objective criteria. Here, we validated all three published graded-prognostic-assessment (GPA)-scores in a subcohort of 882 breast cancer patients with brain metastases in the Brain Metastases in the German Breast Cancer (BMBC) registry. Although all three available GPA-scores were associated with OS, they all show limitations mainly in predicting short-term (below 3 months) survival but also in long-term (above 12 months) survival. We discuss the test performances of all scores in our work and provide evidence how physicians should use them as a tool to select patients for different treatment options.

**Abstract:**

Several scores have been developed in order to estimate the prognosis of patients with brain metastases (BM) by objective criteria. The aim of this analysis was to validate all three published graded-prognostic-assessment (GPA)-scores in a subcohort of 882 breast cancer (BC) patients with BM in the Brain Metastases in the German Breast Cancer (BMBC) registry. The median age at diagnosis of BM was 57 years. All in all, 22.3% of patients (*n* = 197) had triple-negative, 33.4% (*n* = 295) luminal A like, 25.1% (*n* = 221) luminal B/HER2-enriched like and 19.2% (*n* = 169) HER2 positive like BC. Age ≥60 years, evidence of extracranial metastases (ECM), higher number of BM, triple-negative subtype and low Karnofsky-Performance-Status (KPS) were all associated with worse overall survival (OS) in univariate analysis (*p* < 0.001 each). All three GPA-scores were associated with OS. The breast-GPA showed the highest probability of classifying patients with survival above 12 months in the best prognostic group (specificity 68.7% compared with 48.1% for the updated breast-GPA and 21.8% for the original GPA). Sensitivities for predicting 3 months survival were very low for all scores. In this analysis, all GPA-scores showed only moderate diagnostic accuracy in predicting the OS of BC patients with BM.

## 1. Introduction

The incidence of brain metastases (BM) from breast cancer (BC) is increasing and the management and treatment of those patients continue to be a major challenge [1]. BM mostly occur as a late event of metastatic disease after several systemic treatments and their prognosis remains limited [2]. Despite the short median survival times in several cohorts of metastatic BC patients with BM ranging between 7 and 8 months, almost a quarter of patients live longer than 2 years [3]. Although a growing number of therapeutic options for systemic therapy emerge, the management and treatment of BM as well as selecting patients for different therapies remains unsatisfactory. In the past, several prognostic indices have been published in order to help identify patients with good prognosis, eligible for intensive treatment and those with limited prognosis who should be protected from overtreatment with systemic therapy causing side effects. Those patients might be treated better with supportive care in order to help improve their quality of life [4,5]. In 2008, Sperduto et al. established the graded prognostic assessment (GPA)-Score deriving from patients suffering from BM irrespective of their primary tumour localisation [6]. Due to the fact that prognosis differs between different subtypes of primary tumours [7], Sperduto et al. established the disease-specific Breast-GPA score in 2012 deriving from 400 BC patients including prognostic factors such as age, the Karnofsky performance status (KPS) and tumour subtype (Table 1) [8]. An updated version of this score adding the existence of extracranial metastases (ECM) and number of BM to the parameters of the Breast-GPA was published in 2020 in order to increase the test value of the existing Breast-GPA [9]. It has already been described in small cohorts that the Breast-GPA best identifies patients with bad prognosis defined as survival time below 3 months [4,10].

This analysis aimed to examine the performance of the original GPA, the Breast-GPA and the updated Breast-GPA in a large cohort of BC patients with BM in the BMBC registry and to test the accuracy of all scores to predict short survival (defined as below 3 months) or long survival (defined as more than 12 months). 

## 2. Results

### 2.1. Patients’ Characteristics

A total of 882 patients were included in this analysis (Table 2). Patients’ characteristics did not differ between the entire cohort of the BMBC registry and this subset of patients (data not shown).

Median age at the diagnosis of BM was 57 years. Tumour subtypes of the primary tumour were distributed as following: TNBC 22.3% (*n* = 197), Luminal A 33.4% (*n* = 295), Luminal B/HER2 enriched 25.1% (*n* = 221), HER2 positive 19.2% (*n* = 169). All in all, 26.5% of patients had one (*n* = 234), 27.8% had two or three (*n* = 245) and 45.7% had four and more BM (*n* = 403). A further 7.3% of patients (*n* = 64) had leptomeningeal disease. Additionally, 15.6% of patients (*n* = 138) had no ECM. BM were mainly detected by magnetic resonance imaging (MRI) (*n*= 589, 67.1%). Regarding local treatment, the majority of patients received local treatment of BM (86.5%, *n* = 763) either as radiotherapy (*n* = 544), surgery followed by radiotherapy (*n* = 185) or only surgery (*n* = 34). Systemic treatment options were chemotherapy (*n* = 466, 44.9%), endocrine therapy (*n* = 159, 15.3%) or other therapies such as anti-HER2 treatments, bisphosphonates and bevacizumab (*n* = 414, 39.8%) (Table S4, Table S5, Table S6). Most of the patients had a good performance status at diagnosis of BM (KPS 100%: 13.5%, 80–90% 44.6%, 60–70%: 30.3%, 40–50%: 9.1%, 10–30%: 2.6%). 

### 2.2. Survival Analysis

The median OS in our cohort was 8.7 months. Median OS rates at 6-month were 57.4%, at 1-, 2- and 3-year they were 41.3%, 24.3% and 16.6%, respectively.

In univariate analysis, clinical variables like age (categorised in <60/≥60 years), appearance of ECM, KPS, number of BM and biological subtype were all associated with survival (Table 3). Patients 60 years and older had a worse prognosis (median survival of 5 months (95% CI 3.9–5.9) vs. 11.3 months (95% CI 9.6–13.7), Hazard Ratio (HR) 1.53 (1.3–1.77 95% CI, *p* < 0.001). Evidence of ECM at the diagnosis of BM was associated with shorter OS (7.8 months (95% CI 6.4–9.0; HR 1.67, 1.34–2.08 95% CI, *p* < 0.001) vs. 15.6 months (95% CI 9.2–13.6). The number of BM was prognostic with a median OS time of 14.1 months for patients with 1 BM (95% CI 10.3–19.2) followed by 9.7 months for 2–3 BM (95% CI 6.1–12.3) and 6.2 months (95% CI 5.1–7.2) for 4 or more BM (HR for > 4 BM 1.80 (95% CI 1.5–2.16, *p* < 0.001). The tumour subtype of the primary tumour also had an influence on survival times. Patients with TNBC had the shortest median OS with 4.8 months (95% CI 3.8–6.1), followed by Luminal A subtype with 6.0 months (95% CI 5.0–7.9) and HER2 positive (ER-negative) subtype with 12.3 months (95% CI 9.2–17.3), whereas patients with Luminal-B/HER2-enriched subtype had the longest median survival time of 16.0 months (95% CI 13.0–21.7). Consecutively, luminal B/HER2-enriched patients had a significantly lower risk of death compared to patients with other subtypes (HR = 0.43, 95% CI 0.34, 0.53, *p* < 0.001). We also performed an analysis for the progression-free interval after diagnosis of BM and the time from diagnosis of ECM to BM according to breast cancer subtype and found that TNBC patients had the shortest progression-free interval since the first diagnosis of BM of 4.3 months, followed by Luminal A subtype with 5.2 months, HER2 positive subtype with 9.1 months and Luminal B/HER2 enriched subtype with 11.4 months (Table S2, Figure S2). In addition, TNBC patients had the shortest interval between diagnosis of ECM and the first diagnosis of BM of 5.7 months followed by Luminal A subtype with 14.3 months, HER2 positive subtype with 13.8 months and Luminal B/HER2 enriched subtype with 19.3 months. Median survival times in the KPS subgroups differed between 17.9 months (KPS 100%, 95% CI 12.9–22.1) and 2.1 months for KPS 10–30% (1.1–3.0 95% CI). Patients with the lowest KPS (10–30%) had a 4.76 fold increased chance to suffer from an early death (HR 4.76, 95% CI 2.97–7.63, *p* < 0.001) compared to those with a KPS of 100% (Table 3). Multivariate analysis confirmed the association with overall survival for the parameters age ≥60 years, breast cancer subtype, Karnofsky-Performance-Status and number of BM adjusted for ECM (Table S1). 

### 2.3. Prognostic Indices

**Figure 1:**
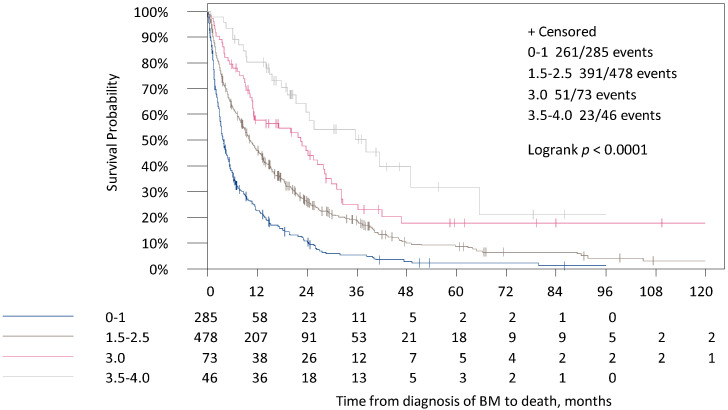
Survival times for the time from diagnosis of BM to death in the four categories of the original GPA Score.

In the original GPA, patients were distributed in four categories depending on their point scores (0–4 p). In total, 32.3% of patients (*n* = 285) were in group 1 (0–1 p) and 5.2% (*n* = 46) in group 4 (3.5–4 p). Median survival times in the four groups were 3.7, 10.1, 22.4 and 38.2 months (Table 4, Figure 1). 

**Figure 2:**
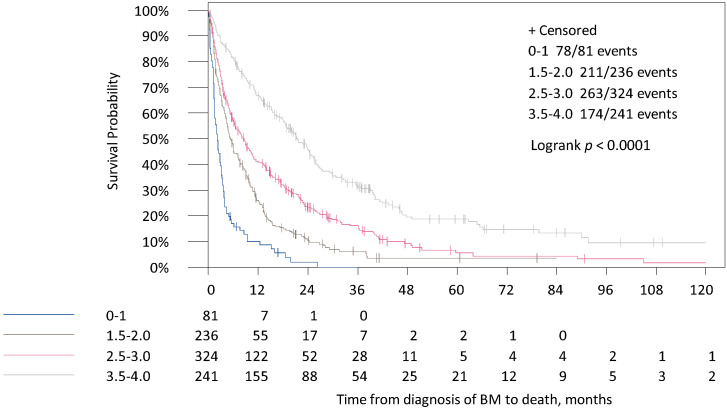
Survival times for the time from diagnosis of BM to death in the four categories of the Breast-GPA Score.

The median survival times between the four categories of the Breast-GPA score were shown to be at 2.2, 5.4, 8.6 and 21.7 months (Table 4, Figure 2). Only 9.2% of patients were included in the worst prognostic group (*n* = 81), whereas 27.3% of patients were included in the best prognostic group (*n* = 241). 

**Figure 3:**
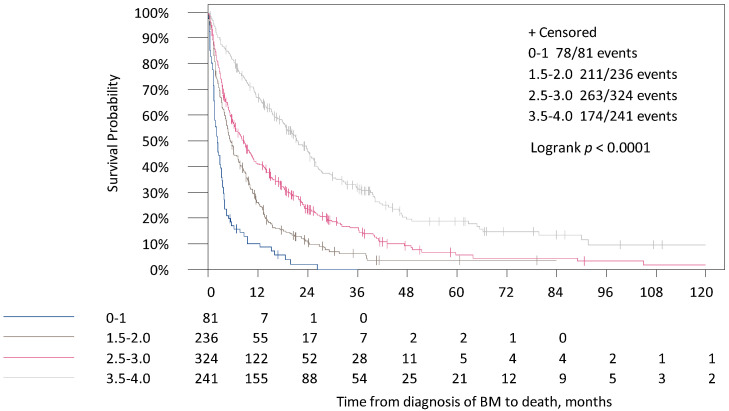
Survival times for the time from diagnosis of BM to death in the four categories of the updated Breast-GPA Score.

In the updated Breast-GPA median survival times variated between 2.7, 5.2, 15.2 and 32.2 months for the four categories (Table 4, Figure 3). All in all, 12.9% of the patients were contributed to the worst prognostic group (*n* = 114) and only 10.1% in the best prognostic group (*n* = 89). All categories defined by all three scores were associated with survival in univariate analysis (*p* < 0.001 for all scores, Table 4). 

### 2.4. Diagnostic Accuracy of GPA Scores

When looking at time-dependent specificities identifying patients with a long-life expectancy (>12 months) the highest category of each score was compared with the lower three categories (Table 5). The Breast-GPA had the highest value of specificity with 68.7% in comparison to the updated Breast-GPA with 48.1% and the original GPA with 21.8%. The time-dependent NPV (the probability of living longer than 12 months with a high point score), was slightly higher for the updated Breast-GPA with 69.1%, compared to the original GPA with 66.3% and the Breast-GPA with 60.2%.

For the identification of patients with a low life expectancy (<3 months) the lowest category was compared with the three higher categories of each score (Table 5). Here, the time-dependent sensitivities for all scores were very low with 24.4% for the original GPA, 11.5% for the updated Breast-GPA and 6.8% for the Breast-GPA. Regarding time-dependent PPV (the probability of living shorter than 3 months with a low point score) the breast specific scores had higher values than the original GPA (62.7% for the updated Breast-GPA and 61.4% for the Breast-GPA vs. 51.3% for the original GPA).

Comparing the Receiver Operating Characteristic (ROC) of the three different scores, the updated Breast-GPA score showed the best results for AUC values of 71.4 for 3 months (95% CI 67.8–75.0) and 74.2 for 12 months (95%-CI 70.9–77.5) in comparison to the Breast-GPA with AUC values of 69.1 for 3 months (95%-CI 65.3–73.0) and 73.0 for 12 months (95%-CI 69.6–76.4) and the original GPA score with AUC values of 70.0 for 3 months (95%-CI 66.3–73.7) and 69.5 for 12 months (95%-CI 66.0–73.1). 

Nonetheless, there were no significant differences between the AUC of all three scores after 12 months (Table 6). On the contrary, the AUC after 3 months was better for the updated Breast-GPA compared with the Breast-GPA (*p* = 0.010, Table 7). In conclusion, there is a lack of discrimination between all scores by gaining AUC results of around 70%. 

## 3. Discussion

Treatment of BM usually involves local and systemic treatment [2]. In almost all cases, local therapy consists of radiotherapy with or without neurosurgery. Despite improved local intracranial control, radiotherapy of the brain did not show improved survival in BC patients and, instead, leads to certain toxicities [11]. Recently, new agents might have also improved survival times in a subset of HER2 positive BC patients with BM [12] and some trials also focus on patients with BM of HER2 negative breast cancer [13]. In order to help to identify patients with good and bad prognosis easily, different scores were developed combining different prognostic parameters [4]. All tests were developed with patients with BM who had received radiotherapy of the brain. In comparison, 14% of patients in our cohort did not receive radiotherapy or local treatment of the brain which might explain the shorter survival times in our cohort in comparison to original cohorts [8]. However, the median OS time of 8.7 months is in the range of published real-world data of BC patients with BM [14]. 

It remains difficult to predict the prognosis of BC patients with BM, although several risk factors have already been identified that are associated with impaired survival. Scores should help to stratify local and systemic treatment according to the patient‘s prognosis. As age, KPS and ECM are associated with survival in patients with BM, these parameters were used to calculate the GPA Score in 2008 [6]. To further improve the test accuracy, the breast-specific scores Breast-GPA and updated Breast-GPA were introduced which include tumour subtype [8,9]. In both breast-specific scores, the luminal-B/HER2 enriched group was established as a separate category. In our analysis, patients with HER2 positive, hormone receptor-positive (triple positive) BC had the best prognosis compared to HER2 positive, hormone receptor-negative and HER2 negative BC. Although triple positivity reveals resistance to HER2-directed treatment in the adjuvant or neoadjuvant setting [15], the prognosis of patients with BM is superior to other subtypes [16,17], thus, supporting the classification as own category in patients with BM. The improved survival of HER2-positive patients is often explained by the possibility of agents suitable for passing the blood–brain barrier [12].

All three GPA scores were associated with OS in our analysis. However, we could show that breast-specific GPA scores which include tumour subtype in the calculation of the score performed slightly better than the original GPA score. However, all scores had a rather low test accuracy in our analysis. The breast-GPA with a specificity of 68.7% and an NPV of 66.3 % for 12-months survival could filter the long-term survivors from the best prognostic group as well as patients with a bad prognosis from the lowest prognostic group (PPV: 61.4%, sensitivity: 6.8%). A high PPV is an important quality factor mainly for the lowest prognostic group in order to prevent patients from getting misleadingly selected in the worst prognosis group and therefore being held from potentially effective therapy [18]. Due to the same reason, the number of patients in the lowest prognostic group should be kept as small as possible [19]. Although the original GPA had the highest sensitivity (24.4%) for predicting 3-months survival, it categorized almost one-third of patients into the lowest category (32.3%). In contrast, the Breast-GPA and the updated Breast-GPA categorized a smaller number of patients into the lowest category (9.2% and 13%). In line with published data, the breast-specific scores had higher PPV and performed slightly better in predicting short survival below 3 months than the original GPA [4]. In addition, the Breast-GPA assigned the highest percentage of patients in the best prognostic group (27.3%) in comparison to the updated Breast-GPA (10.1%) and the original GPA (5.2%). This could explain the shorter median survival time for the breast-GPA patients of the best prognostic group with 21.7 months (original GPA 38.2 and updated Breast-GPA 32.2 months). The breast-GPA also had the highest specificity (68.7%) in identifying patients with a long life expectancy (>12 months). It needs to be discussed whether the best prognostic group should separate only a minority of patients with an excellent prognosis of more than 3 years or a larger group with also a very good prognosis of around 2 years for selecting therapy options. 

## 4. Materials and Methods

The BMBC registry is a multicentre trial evaluating clinical data of BC patients with BM run by the German Breast Group (GBG), the Translation Research Board and the Breast Study Group of the Working Group Gynaecologic Oncology Germany (AGO-Trafo and AGO-B) and the University Medical Center Hamburg, Germany. Patients were identified retrospectively as well as prospectively if they had a diagnosis of BM based on appropriate imaging and/or histological findings since the year 2000 and a history of BC. Patients were excluded if they had a history of other malignant diseases, no histological verification of the diagnosis of BC, a history of neurologic disease or leptomeningeal disease without solid BM. By August 2019, 105 study sites had documented clinical data of 2589 patients. In sum, 1158 patients (44.73%) were treated in a university setting, 1431 (55.27%) in a non-university setting. All participating study sites were either located in departments of gynaecology or medical oncology. The BMBC registry was approved by all local ethics committees. For 882 (34%) from overall 2589 patients, all three GPA scores could be determined and therefore were considered eligible for this study.

### 4.1. Calculation of GPA Scores

In the original GPA score, four groups reflecting median survival times were calculated on the basis of the prognostic factors KPS, number of BM, ECM and age at the first diagnosis of BM. In the Breast-GPA number of BM and ECM were replaced by tumour subtype and also four median survival groups were estimated. In the updated version of the Breast-GPA score presence of ECM was added to the parameters already included in the Breast-GPA Score and four survival groups were calculated. For parameters and calculation of the GPA Scores, see Table 1.

### 4.2. Biological Subtype and ECOG/Karnofsky Performance Status

In order to calculate the disease-specific GPA scores tumour subtypes had to be redefined as HER2 positive including only estrogen receptor (ER) and/or progesterone receptor (PR) negative and HER2 positive tumours and Luminal B-HER2 enriched including HER2 positive and ER and/or PR positive tumours. Luminal A was defined as ER-positive, HER2 negative and triple-negative as ER-negative and HER2 negative. 

ECOG/Karnofsky performance status was categorized in 0 (100%), 1 (80–90%), 2 (60–70%), 3 (40–50%), 4 (10–30%) and unknown.

### 4.3. Statistical Analysis

Continuous data were summarized using the number of available data, mean, standard deviation (SD), median, minimum and maximum for each group. Categorical and ordinal data were summarized using the number and percentage of patients in each group. 

Further, Kaplan–Meier curves and the median OS time with the corresponding 95% confidence interval (CI) were determined to assess the association of the OS with the survival times of several factors (age at first diagnosis of BM, number of BM, biological subtype, KPS, appearance of ECM) and of the three prognostic scores. OS was defined as the time interval from the first diagnosis of BM to death due to any reason. Differences in the survival curves were tested by the log-rank test. All reported *p*-values are two-sided, and the significance level was set to 0.05. Confidence intervals symmetrically cover 95%. 

The data were analyzed using SAS® (Statistical Analysis Software) version 9.4 (SAS Institute Inc., Cary, NC, USA)with SAS Enterprise Guide Version 7.1 on Microsoft Windows 10 Enterprise (Microsoft Corporation, Redmond, WA, USA). The diagnostic accuracy of the Breast-GPA, the updated Breast-GPA and the original GPA score was described by time-dependent sensitivities, specificities, positive and negative predictive values (PPV; NPV) at the time points after 3 months to identify a short life expectancy and after 12 months to identify a long life expectancy for the selected cut-off values. For the identification of a short life expectancy (<3 months) a high score of sensitivity and PPV was defined as the best factors for excellent test quality, whereas for the identification of a long life expectancy (>12 months), a high specificity and high NPV were defined as best factors for excellent test quality. Cut-offs for short- and long-term survival were used in order to help identify patients both eligible for extensive treatment and spare patients with worse outcomes from overtreatment. Additionally, the corresponding 95%-CI of these time-dependent measures were determined. 

Furthermore, as a measure of accuracy, the areas under the time-dependent Receiver Operating Characteristic (ROC) curves (AUC) were determined. In the time-dependent ROC curves, the sensitivities were plotted against the specificities for different cut-off values of the original score, the Breast-GPA and the updated Breast-GPA at the time of 3 and 12 months. 

The analyses of the described time-dependent measures were performed using R from the R Foundation for Statistical Computing, Vienna, Austria (version 3.6.0), particularly the R package time ROC by P. Blanche (version 0.3) [20]. 

## 5. Conclusions

In summary, we could show in a real-world cohort of BC patients with BM that although all GPA-Scores were associated with overall survival, the addition of disease-specific parameters resulted in better test accuracy. However, adding those parameters did not improve the accuracy as much as expected. All currently available prognostic scores show limitations mainly in predicting short-term (below 3 months) survival but also in long-term (above 12 months) survival and, thus, should be employed carefully by physicians when being used for further therapy decisions. Further studies should focus on the identification of biomarkers that might help to improve estimating the prognosis of BC patients with BM. 

## Figures and Tables

**Table 1:** Calculation of original graded-prognostic-assessment (GPA), Breast-GPA and updated Breast-GPA.

GPA	0 points	0.5 points	1.0 points	
Karnofsky	≤60	70–80	90–100	
Number of BM	≥4	2–3	1	
ECM	yes		no	
Age	≥60	50–59	<50	
group 1: 0–1P; 2:1.5–2.5P; 3: 3.0P; 4: 3.5–4.0P				
Breast-GPA	0 points	0.5 points	1.0 points	1.5 points
Karnofsky	≤50	60	70–80	90–100
Subtype	TNBC	n/a	LumA	HER2
Age	≥60	<60	n/a	n/a
group 1:0–1 P.; 2:1.5–2.0 P.;3: 2.5–3.0 P.; 4:3.5–4.0 P.				
Updated Breast-GPA	0 points	0.5 points	1.0 points	1.5 points
Karnofsky	≤60	70–80	90–100	n/a
Subtype	TNBC	n/a	LumA	HER2, LumB
Age	≥60	<60	n/a	n/a
Number of BM	>1	=1	n/a	n/a
ECM	yes	no		
group 1: 0–1P.; 2: 1.5–2.0 P.; 3:2.5–3.0 P.; 4: 3.5–4.0 P.				

**Table 2:** Patients’ characteristics (*n* = 882).

Parameter	Category	Number (%)
Age at first diagnosis of BC, years	Median	53.0
	range	20.0, 87.0
	missing	0
Age at diagnosis of BM, years	Median	57.0
	range	25.0, 90.0
	missing	0
Histological tumour type	ductal or ductal-lobular-invasive	691 (78.8)
	lobular-invasive	60 (6.8)
	other	126 (14.4)
	missing	5
Biological subtype (according to Sperduto 2012)	TNBC (HR−/HER2−)	197 (22.3)
	Luminal A (HR+, HER2−)	295 (33.4)
	Luminal B/HER2 enriched (HR+, HER2+)	221 (25.1)
	HER2 (HR−, HER2+)	169 (19.2)
	missing	0
KPS at diagnosis of BM	100%	119 (13.5)
	80–90%	393 (44.6)
	60–70%	267 (30.3)
	40–50%	80 (9.1)
	10–30%	23 (2.6)
	missing	0
Number of BM	1	234 (26.5)
	2–3	245 (27.8)
	≥4	403 (45.7)
	missing	0
Diagnostic method to detect BM	only clinical	9 (1.0)
	CT w/o clinical	209 (23.8)
	MRI w/o clinical	589 (67.1)
	CT and MRI w/o clinical	71 (8.1)
	missing	4
Local treatment of BM	Surgery only	34 (4.5)
	Radiotherapy only	544 (71.3)
	Surgery and radiotherapy	185 (24.2)
	missing	119
ECM	no	138 (15.6)
	yes	744 (84.4)
Leptomeningeal metastases	no	814 (92.7)
	yes	64 (7.3)
	missing	4

**Table 3:** Median overall survival after the diagnosis of brain metastases (BM) (univariate analysis).

Parameter	Category	Median Survival (months)	95%-CI	HR	95% CI	*p*-Value
Age at diagnosis of BM, binary	<60	11.3	(9.6, 13.7)			
	≥60	5	(3.9, 5.9)	1.53	(1.32, 1.77)	<0.001
ECM	no	15.6	(10.8, 23.8)			
	yes	7.8	(6.4, 9.0)	1.67	(1.34, 2.08)	<0.001
Number of BM	1	14.1	(10.3, 19.2)			
	2–3	9.7	(6.1, 12.3)	1.34	(1.09, 1.65)	0.005
	≥4	6.2	(5.1, 7.2)	1.80	(1.50, 2.16)	<0.001
Biological subtype (acc. to Sperduto 2012)	TNBC	4.8	(3.8, 6.1)			
	Luminal A (HR+, HER2-)	6.0	(5.0, 7.9)	0.79	(0.65, 0.95)	0.014
	Luminal B (HR+, HER2+)	16.0	(13.0, 21.7)	0.43	(0.34, 0.53)	<0.001
	HER2 (HR-, HER2+)	12.3	(9.2, 17.3)	0.49	(0.39, 0.62)	<0.001
KPS	100%	17.9	(12.9, 22.1)			
	80–90%	13.0	(10.8, 15.1)	1.26	(0.99, 1.60)	0.057
	60–70%	4.4	(3.5, 5.2)	2.28	(1.78, 2.92)	<0.001
	40–50%	2.9	(2.2, 3.9)	3.66	(2.68,5.00)	<0.001
	10–30%	2.1	(1.1, 3.0)	4.76	(2.97, 7.63)	<0.001

**Table 4:** Median survival according to the three GPA scores after the diagnosis of BM.

Parameter	Group	Points	Number of Patients	Median Survival (months)	HR	95%-CI	*p*-Value
Original GPA	1	0–1	285	3.7			
	2	1.5–2.5	478	10.1	0.53	(0.46, 0.63)	<0.001
	3	3.0	73	22.4	0.34	(0.25, 0.46)	<0.001
	4	3.5–4.0	46	38.2	0.21	(0.14, 0.32)	<0.001
Breast-GPA	1	0–1	81	2.2			
	2	1.5–2.0	236	5.4	0.47	(0.36, 0.61)	<0.001
	3	2.5–3.0	324	8.6	0.31	(0.24, 0.41)	<0.001
	4	3.5–4.0	241	21.7	0.17	(0.13, 0.23)	<0.001
updated Breast-GPA	1	0–1	114	2.7			
	2	1.5–2.0	327	5.2	0.63	(0.50, 0.78)	<0.001
	3	2.5–3.0	352	15.2	0.29	(0.23, 0.37)	<0.001
	4	3.5–4.0	89	32.2	0.15	(0.11, 0.21)	<0.001

**Table 5:** Diagnostic accuracy of the original GPA, the Breast-GPA and updated Breast-GPA score at the time of 12 months for a cut off value of 3, comparing the highest category versus the three lower categories of each score.

GPA-Score	Timepoint	Time-Dependent Sensitivity (%) (95%-CI)	Time-Dependent Specificity (%) (95%-CI)	Time-Dependent PPV (%) (95%-CI)	Time-Dependent NPV (%) (95%-CI)
Original GPA	12 months	92.2 (89.9, 94.6)	21.8 (17.4, 26.2)	62.6 (59.1, 66.1)	66.3 (57.7, 75.0)
Breast-GPA	12 months	68.1 (64.0, 72.2)	68.7 (63.8, 73.7)	75.6 (71.6, 79.6)	60.2 (55.4, 65.0)
updated Breast GPA	12 months	84.8 (81.7, 88.0)	48.1 (42.8, 53.4)	69.9 (66.2, 73.6)	69.1 (63.2, 74.9)

**Table 6:** Diagnostic accuracy of the original GPA, the Breast-GPA and updated Breast-GPA score at the time of 3 months for a cut off value of 1, comparing the lowest category versus the three higher categories of each score.

GPA-Score	Time Point	Time-Dependent Sensitivity (%) (95%-CI)	Time-Dependent Specificity (%) (95%-CI)	Time-Dependent PPV (%) (95%-CI)	Time-Dependent NPV (%) (95%-CI)
Original GPA	3 months	24.4 (18.9, 29.9)	91.6 (89.4, 93.7)	51.3 (42.0, 60.6)	76.9 (73.9, 79.9)
Breast-GPA	3 months	6.8 (3.6, 10.1)	98.4 (97.5, 99.4)	61.4 (42.7, 80.2)	74.4 (71.4, 77.3)
updatedBreast-GPA	3 months	11.5 (7.4, 15.6)	97.5 (96.3, 98.7)	62.7 (48.3, 77.2)	75.2 (72.2, 78.1)

**Table 7:** Area under the time-dependent Receiver Operating Characteristic (ROC) curve (AUC) with the corresponding 95%-CI for all three GPA-Scores for two different time points (3 and 12 months).

GPA-Score	Time Point	AUC (%) (95%-CI)	Comparison	*p*-Value
Original GPA	3 months	70.0 (66.3, 73.7)		
Original GPA	12 months	69.5 (66.0, 73.1)		
Breast-GPA	3 months	69.1 (65.3, 73.0)	Breast-GPA vs. original GPA	0.698
Breast-GPA	12 months	73.0 (69.6, 76.4)		0.086
updated Breast-GPA	3 months	71.4 (67.8, 75.0)	Breast-GPA vs. updated Breast-GPA	0.010
updated Breast-GPA	12 months	74.2 (70.9, 77.5)		0.176

## Data Availability

Data is contained within the article or Supplementary Material.

## Supplementary Materials

The following are available online at https://www.mdpi.com/2072-6694/13/4/844/s1. Table S1: Multivariate Cox regression analysis of the time from BM to death, Table S2: Progression free Survival according to Breast Cancer subtype, Table S3: Distribution of the subtype (according to Sperduto 2012) in patients with meningeosis carcinomatosa (N = 252 of overall 2589 patients), Table S4: Local treatment according to subtype (Sperduto 2012), Table S5: Distribution of BC treatment between different subtypes after the diagnosis of BM, Table S6: specification of BC treatment after the diagnosis of BM, Figure S1: Overall Survival for Leptomeningeal disease and solid BM vs. only solid BM, Figure S2: Time from first diagnosis of BM to progress
