# Supplementary material for: Predicting Prognosis of Breast Cancer Patients with Brain Metastases in the BMBC Registry—Comparison of Three Different GPA Prognostic Scores
Source: Cancers (Basel). 2021 Feb 17;13(4):844. doi: 10.3390/cancers13040844 (PMC7922206; doi:10.3390/cancers13040844)
Supplement: Supplementary file 1 [file cancers-13-00844-s001.pdf]

cancers

*Article*

# Predicting Prognosis of Breast Cancer Patients with Brain Metastases in the BMBC Registry—Comparison of Three Different GPA Prognostic Scores

## Supplementary Materials

**Table S1.** Multivariate Cox regression analysis of the time from BM to death.

| Parameter | Category | Hazard Ratio | 95% CI | *p*-Value |
|---|---|---|---|---|
| Biological subtype (acc. to Sperduto 2012) | TNBC | | | <0.001 |
| | Luminal A (HR+/HER2-) | 0.721 | (0.592, 0.878) | 0.001 |
| | Luminal B (HR+/HER2+) | 0.433 | (0.348, 0.539) | <0.001 |
| | HER2 (HR-/HER2+) | 0.451 | (0.358, 0.570) | <0.001 |
| Age at diagnosis of BM, binary | <60 | | | |
| | ≥60 | 1.44 | (1.24, 1.68) | <0.001 |
| KPS | 100% | | | <0.001 |
| | 80-90% | 1.06 | (0.833, 1.35) | 0.636 |
| | 60-70% | 1.85 | (1.44, 2.38) | <0.001 |
| | 10-50% | 2.86 | (2.12, 3.86) | <0.001 |
| Number of BM | 1 | | | <0.001 |
| | 2-3 | 1.39 | (1.13, 1.72) | 0.002 |
| | ≥4 | 1.68 | (1.38, 2.03) | <0.001 |
| ECM at BM diagnosis | no | | | |
| | yes | 1.23 | (.981, 1.55) | 0.072 |

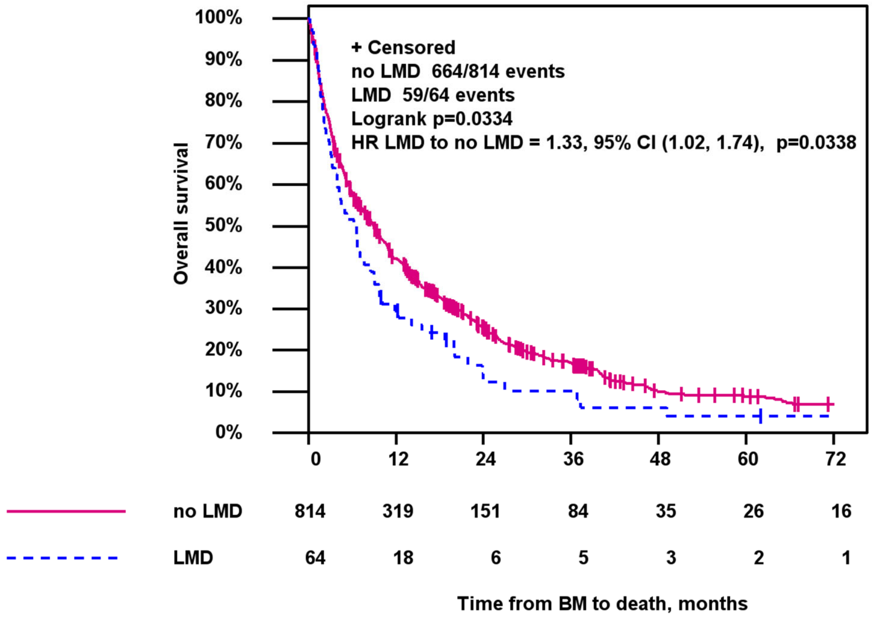

**Figure S1.** Overall Survival for Leptomeningeal disease and solid BM vs. only solid BM.

**Table S2.** Progression free Survival according to Breast Cancer subtype.

| Survival | Subtype | Median (95% CI) |
|---|---|---|
| PFS | TNBC | 4.3 (3.5, 5.1) |
| | Luminal A (HR+/HER2-) | 5.2 (4.2, 6.4) |
| | Luminal B (HR+/HER2+) | 11.4 (9.2,13.4) |
| | HER2 (HR-/HER2+) | 9.1 (7.7,11.0) |

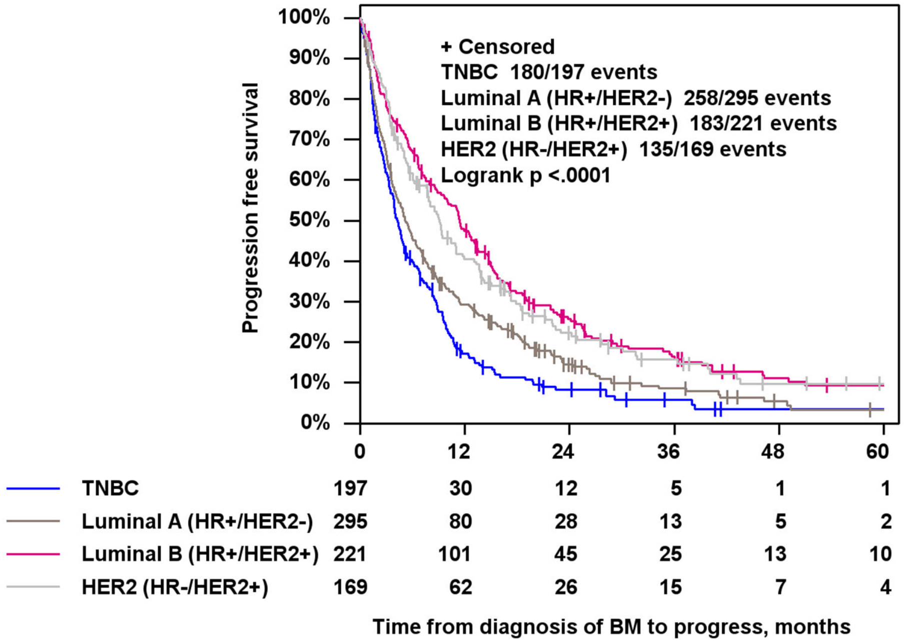


**Figure S2.** Time from first diagnosis of BM to progress.

**Table S3.** Distribution of the subtype (according to Sperduto 2012) in patients with meningeosis carcinomatosa ($n$ = 252 of overall 2589 patients).

| Subtype According to Sperduto 2012 | N(%) (*n* =252) |
|---|---|
| *TNBC* | 52 (23.74) |
| *Luminal A* | 82 (37.44) |
| *Luminal B* | 49 (22.37) |
| *HER2* | 36 (16.44) |
| *Missing* | 33 |

**Table S4.** Local treatment according to subtype (Sperduto 2012).

| Local Treatment | Subtype (Sperduto 2012) | Number (%) |
|---|---|---|
| Surgery only | TNBC (HR-/HER2-) | 4 ( 2.4) |
| | Luminal A (HR+, HER2 -) | 14 (5.6) |
| | Luminal B/HER2 enriched | 9 (4.6) |

| | | |
|---|---|---|
| | (HR +, HER2 +) | |
| | HER2 (HR-/HER2+) | 7 (4.6) |
| Radiotherapy only | TNBC (HR-/HER2-) | 120 (73.2) |
| | Luminal A (HR+, HER2 -) | 186 (74.1) |
| | Luminal B/HER2 enriched (HR +, HER2 +) | 133 (68.2) |
| | HER2 (HR-/HER2+) | 105 (68.6) |
| Surgery and Radiotherapy | TNBC (HR-/HER2-) | 40 (24.4) |
| | Luminal A (HR+, HER2 -) | 51 (27.2) |
| | Luminal B/HER2 enriched (HR +, HER2 +) | 53 (27.2) |
| | HER2 (HR-/HER2+) | 41 (26.8) |
| Missing | TNBC (HR-/HER2-) | 33 |
| | Luminal A (HR+, HER2 -) | 41 |
| | Luminal B/HER2 enriched (HR +, HER2 +) | 26 |
| | HER2 (HR-/HER2+) | 16 |

**Table S5.** Distribution of BC treatment between different subtypes after the diagnosis of BM.

| | **TNBC** *n* = 81 N(%) | **Luminal A (HR+/HER2-)** *n* = 151 N(%) | **Luminal B (HR+/HER2+)** N(%) *n* = 131 | **HER2 (HR-/HER2+)** *n* = 94 N(%) | **Overall** *n* = 457 * N(%) |
|---|---|---|---|---|---|
| Chemotherapy | 123 (76.9) | 143 (46.6) | 118 (33.2) | 82 (37.8) | 466 (44.9) |
| endocrine therapy | 3 ( 1.9) | 94 (30.6) | 57 (16.1) | 5 ( 2.3) | 159 (15.3) |
| Targeted therapy | 34 (21.3) | 70 (22.8) | 180 (50.7) | 130 (59.9) | 414 (39.8) |

**Table S6.** specification of BC treatment after the diagnosis of BM.

| | **TNBC** *n* = 81 N(%) | **Luminal A (HR+/HER2-)** *n* = 151 N(%) | **Luminal B (HR+/HER2+)** N(%) *n* = 131 | **HER2 (HR-/HER2+)** *n* = 94 N(%) | **Overall** *n* = 457 * N(%) |
|---|---|---|---|---|---|
| Anthracycline | 12 (7.5) | 32 (10.4) | 12 (3.4) | 3 (1.4) | 59 (5.7) |
| Taxane based | 8 (5.0) | 16 (5.2) | 20 (5.6) | 13 (6.0) | 57 (5.5) |
| Taxane + Anthracycline | 32 (20.0) | 43 (14.0) | 39 (11.0) | 25 (11.5) | 139 (13.4) |
| Other chemotherapy | 71 (44.4) | 52 (16.9) | 47 (13.2) | 41 (18.9) | 211 (20.3) |
| Tamoxifen | | 10 (3.3) | 7 (2.0) | 2 (0.9) | 19 (1.8) |
| Aromatase Inhibitor | 2 (1.3) | 53 (17.3) | 30 (8.5) | 2 (0.9) | 87 (8.4) |
| GnRH-Analoga | | 8 (2.6) | 3 (0.3) | | 11 (1.1) |
| Other HT | 1 (0.6) | 23 (7.5) | 17 (4.8) | 1 (0.5) | 42 (4.0) |
| Trastuzumab | 2 (1.3) | 6 (2.0) | 64 (18) | 44 (20.3) | 116 (1.2) |
| Trastuzumab+Pertuzumab | | 2 (0.7) | 15 (4.2) | 7 (3.2) | 24 (2.3) |
| Lapatinib | 1 (0.6) | 6 (2.0) | 43 (12.1) | 38 (17.5) | 88 (8.5) |
| T-DM1 | | 3 (1.0) | 29 (8.2) | 26 (12.0) | 58 (5.6) |
| Everolimus | | 5 (1.6) | 1 (0.3) | | 6 (0.6) |
| Bisphophonates | 8 (5.0) | 18 (5.9) | 15 (4.2) | 8 (3.7) | 49 (4.7) |
| Denosumab | 13 (8.1) | 16 (5.2) | 9 (2.5) | 3 (1.4) | 41 (3.9) |
| Bevacizumab | 10 (6.3) | 14 (4.6) | 2 (0.6) | 3 (1.4) | 29 (2.8) |

| | | | |
|---|---|---|---|
| Other | 2 (0.6) | 1 (0.5) | 3 (0.3) |
